# Hi-C Technology Reveals Actionable Gene Fusions and Rearrangements in Diffuse Large B-Cell Lymphoma Unidentified by Conventional FISH

**DOI:** 10.3390/genes16091093

**Published:** 2025-09-16

**Authors:** Sichen Liang, Candice Ament, Melanie Klausner, Victoria Stinnett, Laura Morsberger, Jen Ghabrial, William Middlezong, Anthony D. Schmitt, Alex R. Hastie, Ying S. Zou

**Affiliations:** 1 Department of Obstetrics and Gynecology, Peking University People’s Hospital, Beijing 100044, China; sliang36@jh.edu; 2 Department of Pathology, Johns Hopkins University School of Medicine, Baltimore, MD 21205, USA; cament1@jhmi.edu (C.A.); mhardy22@jhmi.edu (M.K.); vlloyd3@jhmi.edu (V.S.); lmorsber@jhmi.edu (L.M.); jghabri1@jh.edu (J.G.); wmiddle2@jhu.edu (W.M.); 3 Arima Genomics, Carlsbad, CA 92011, USA; anthony@arimagenomics.com (A.D.S.); alex.hastie@arimagenomics.com (A.R.H.)

**Keywords:** Hi-C, lymphoma, FISH, gene rearrangements, 3D genomics, gene fusions, DLBCL

## Abstract

Background/Objectives: Fluorescence in situ hybridization (FISH) is a standard diagnostic tool for detecting gene fusions and rearrangements in lymphomas but is limited by incomplete genomic coverage, dependence on predefined probes, and difficulty identifying atypical or noncanonical fusion partners. These constraints often result in inconclusive diagnoses in complex lymphoma cases. This study evaluates a novel Hi-C-based sequencing assay from formalin-fixed paraffin-embedded (FFPE) samples to detect clinically significant gene fusions and rearrangements in cases where conventional FISH was inconclusive or expected biomarkers were not detected. Methods: Five diffuse large B-cell lymphoma cases with previously atypical gene fusions or rearrangements by FISH were analyzed using both standard FISH and a Hi-C-based lymphoma assay. Standard FISH was performed using break-apart probes targeting *MYC*, *BCL2*, and *BCL6*, and dual-fusion probes targeting *IGH*::*MYC* and *IGH*::*BCL2*. The Hi-C assay utilized high-resolution sequencing of FFPE tissue to map chromatin interactions and identify structural variations across the genome and assessment of their clinical relevance. Results: In this series of five lymphoma cases, Hi-C detected additional structural variants beyond those identified by FISH. It identified typical and atypical translocation partners of key oncogenes (*MYC*, *BCL2*, *BCL6*), cryptic breakpoints, and novel genomic events, including *TP53* loss, *KMT2A* amplification, and complex rearrangements, which were undetectable by FISH. The Hi-C assay’s whole-genome coverage enabled comprehensive profiling. Conclusions: The Hi-C-based lymphoma assay offers a transformative diagnostic tool, overcoming FISH limitations by providing unbiased, high-resolution detection of structural variations. This approach enhances diagnostic accuracy and supports personalized therapeutic strategies in lymphoma management, warranting further validation for clinical adoption.

## 1. Introduction

Diffuse large B-cell lymphoma (DLBCL) is the most common subtype of non-Hodgkin lymphoma (NHL), accounting for approximately 30–40% of all lymphoma cases [1]. It is an aggressive malignancy arising from mature B-cells, characterized by diffuse proliferation of large lymphoid cells with a high mitotic rate. It arises from genetic and epigenetic alterations that disrupt B-cell development, leading to uncontrolled proliferation and survival [1]. Recurrent gene fusions and chromosomal rearrangements involving *MYC*, *BCL2*, and *BCL6* are hallmark genetic events in DLBCL and are defining features of specific DLBCL subtypes and high-grade B-cell lymphomas (HGBCL), as per the World Health Organization (WHO) 2016 and 2022 classifications [2,3]. They are pivotal in its pathogenesis, driving oncogenesis and serving as diagnostic, prognostic, and therapeutic markers. These genetic alterations enable precise subtype classification, guide risk stratification, and inform treatment decisions, such as R-CHOP, targeted therapies, and clinical trials [4,5,6,7,8,9].

While recurrent gene fusions and chromosomal rearrangements can result from translocations, inversions, or deletions, they frequently involve translocations that juxtapose oncogenes with regulatory elements, such as the immunoglobulin heavy chain locus (*IGH*) on chromosome 14q32, or other immunoglobulin loci (*IGK*, *IGL*) [8,9,10,11]. This juxtaposition leads to oncogenesis by dysregulating key cellular processes, such as proliferation, apoptosis, and differentiation. For example, the *IGH*::*MYC* fusion [t(8;14)(q24;q32)] drives overexpression of the MYC proto-oncogene, promoting uncontrolled cell proliferation and genomic instability [10]. Similarly, *IGH*::*BCL2* [t(14;18)(q32;q21)] leads to overexpression of the anti-apoptotic BCL2 protein, enhancing tumor cell survival [12]. In double-hit lymphomas, combined dysregulation of *MYC* and *BCL2* (often with *BCL6*) results in synergistic oncogenesis, characterized by rapid tumor growth and resistance to apoptosis [13]. These gene fusions or rearrangements are critical for diagnosis, distinguishing DLBCL subtypes (e.g., germinal center B-cell-like) and HGBCL, and aid prognosis, as HGBCL are associated with poor outcomes [2,3]. Therefore, precise detection of gene fusions and rearrangements is essential for accurate diagnosis, precise classification, prognostic assessment, and personalized treatment of DLBCL patients.

Fluorescence in situ hybridization (FISH) is a cornerstone diagnostic tool in lymphoma, serving as the gold standard for detecting specific gene rearrangements, including *MYC*, *BCL2*, and *BCL6*, as well as gene fusions such as *IGH*::*MYC* and *IGH*::*BCL2* [14]. Utilizing break-apart and dual-fusion probes, FISH achieves high specificity in identifying these alterations in DLBCL and HGBCL, enabling precise diagnosis, subtype classification, and detection of high-risk double-hit/HGBCL [15]. Its compatibility with formalin-fixed paraffin-embedded (FFPE) tissue make it indispensable in clinical settings. However, FISH’s targeted approach requires prior knowledge of genetic targets, and sequential probing can be costly and time- and tissue-intensive. Atypical breakpoints, noncanonical fusion partners (e.g., *IGK*::*MYC*), and technical challenges with FFPE samples may reduce sensitivity, potentially missing clinically actionable or significant variants [16,17].

Hi-C, a high-throughput three-dimensional (3D) genomics technique derived from chromosome conformation capture (3C), maps genome-wide chromatin interactions to reveal the spatial organization of the genome [18,19,20,21]. By capturing pairwise interactions between DNA regions, Hi-C detects structural variants, including translocations, inversions, and deletions, with unprecedented resolution and comprehensiveness [22,23,24,25,26]. In lymphoma diagnostics, particularly for DLBCL and HGBCL, a Hi-C-based lymphoma assay enables diagnosis-agnostic detection of gene fusions of *IGH*::*MYC* and *IGH*::*BCL2*, and rearrangements of *MYC*, *BCL2*, and *BCL6*, in a single assay [27]. It is capable of detecting atypical breakpoints and identifying noncanonical fusion partners. With higher sensitivity than FISH, which is the current gold standard for detecting *MYC*, *BCL2*, and *BCL6* rearrangements, the Hi-C assay overcomes limitations of targeted approaches. This study evaluates the Hi-C-based lymphoma assay in five atypical lymphoma cases, demonstrating its superior ability to detect clinically significant gene fusions and rearrangements missed by conventional FISH studies, thereby providing critical insights into its diagnostic and therapeutic potential.

## 2. Materials and Methods

### 2.1. Sample Selection

Five lymphoma cases were selected from the Johns Hopkins Hospital (JHH) Clinical Cytogenetic Lab database, from 1 January 2022, to 30 May 2025. These cases were chosen based on atypical or inconclusive FISH results for JHH standard B-cell lymphoma panels targeting *MYC*, *BCL2*, and *BCL6* rearrangements. Selection criteria included atypical rearrangements/noncanonical fusion partners, or negative FISH results despite high clinical suspicion for rearrangements, enabling evaluation of the Hi-C-based lymphoma assay’s ability to detect novel or cryptic genetic alterations for improved diagnostic and therapeutic precision.

### 2.2. FISH Analysis

FISH was performed using JHH standard B-cell lymphoma panels on FFPE tumor specimens, targeting *MYC* [8q24], *BCL2* [18q21], *BCL6* [3q27], and *IGH* [14q32] to detect rearrangements and specific fusions, including *IGH*::*MYC* [t(8;14)(q24;q32)] and *IGH*::*BCL2* [t(14;18)(q32;q21)], in suspected DLBCL and HGBCL cases. Break-apart probes identified rearrangements of *MYC*, *BCL2*, and *BCL6*, while dual-fusion probes confirmed the two specific translocations of *IGH*::*MYC* and *IGH*::*BCL2* (Probes for *MYC*, *IGH*::*MYC*, and *IGH*::*BCL2* were from ZytoVision Inc., Bremerhaven, Germany and *BCL2* and *BCL6* probes were from Abbott Molecular, Inc., Des Plaines, IL, USA), used according to the manufacturer’s protocol as previously described [28]. *TP53* [17p13] and *KMT2A* [11q23] (Abbott Molecular, Inc., Des Plaines, IL, USA) were used for follow-up FISH testing. A total of 100 interphase nuclei per probe were visually evaluated with fluorescence microscopy by two technologists scoring blinded from each other using a Zeiss Axioscope system. For break-apart probes targeting *MYC*, *BCL2*, and *BCL6*, a FISH signal pattern of 0-3 fusion (F), 1 red (R), and 1 green (G) (0-3F1R1G) with distinct separation of red and green signals indicated a typical rearrangement of *MYC*, *BCL2*, or *BCL6* in a cell. Other split signal patterns with unequal numbers of red and green signals suggested atypical rearrangements. For dual-fusion probes detecting *IGH*::*MYC* and *IGH*::*BCL2* translocations, a FISH signal pattern of 2-3 fusion (F), 1 red (R), and 1 green (G) (2-3F1R1G) confirmed fusions of *IGH* with *MYC* or *BCL2*. Other fusion signal patterns with unequal numbers of red and green signals indicated atypical fusions/rearrangements. Amplification was defined as the presence of more than four copies of the target gene. A positive rearrangement for break-apart FISH probes was indicated by ≥10% of nuclei showing abnormal signals (e.g., split signals), while ≥15% of nuclei displaying abnormal signals (e.g., fusion signals) indicated a positive gene fusion for dual-fusion probes, consistent with established laboratory cutoffs and clinical guidelines.

### 2.3. Hi-C-Based Lymphoma Assay

The Hi-C assay employed a modified protocol optimized for FFPE lymphoma sam-ples. FFPE tissue from unstained slides (Supplemental Table S1) were processed using the Arima HiC+ for FFPE kit (Product Number A311038, Arima Genomics, Carlsbad, CA, USA) as per manufacturer protocols [27]. High-resolution paired-end sequencing was conducted on an Element Biosciences, Aviti platform to capture 3D chromatin long-range interactions across the genome. Sequencing captured approximately 100 million read pairs. A custom bioinformatics pipeline, incorporating structural variation detection algorithms, was used to identify translocations, amplifications, deletions, and complex rearrangements, with breakpoints and fusion partners annotated against the GRCh38 reference genome. Data was processed using Arima-SV-Pipeline (v1.3; https://github.com/ArimaGenomics/Arima-SV-Pipeline, accessed on 10 June 2025), comprising HiCUP (v0.8.0) [29] to calculate quality control metrics and perform read alignment and filtering, hic_breakfinder (v1.0, Dixon) [21,26] and EagleC (v0.1.9) [30] to call rearrangement breakpoints, and Juicer Tools (v1.6) (Durand) to produce multi-resolution Hi-C matrices from mapped and filtered read pairs.

Where hic_breakfinder and EagleC identified breakpoints, we prioritized EagleC’s calls for the merged dataset, followed by rigorous manual review for validation. No breakpoints were called based solely on hic_breakfinder. For the rearrangement breakpoint calls, we manually reviewed the Hi-C matrix data for lymphoma-associated genes in this call set. We confirmed that all breakpoints align with precise Hi-C signal boundaries, supported by evidence of proximity-based signal decay corresponding to the breakpoint strand orientation. Details of the manual review process have been previously described [27]. Copy number was calculated from Hi-C data at 25 kbp resolution using the calculate-cnv and segment-cnv utilities from NeoLoopFinder (v0.4.3-r2) with default parameters [27,31]. The Hi-C Lymphoma assay was conducted using Arima Genomics Hi-C kits (Carlsbad, CA, USA). The assay captures whole-genome interactions, preserving spatial gene positioning, and assesses 417 lymphoma-associated genes, selected based on established guidelines and literature, to investigate lymphoma pathogenesis (Supplemental Table S2). After SV calling, all rearrangements within 1.5 Mb were manually curated for accuracy and impact on genes affected. Data were analyzed for rearrangements, fusions, copy number changes, and deletions within 1 Mb of target genes.

### 2.4. Data Analysis

FISH and Hi-C-based lymphoma assay results were compared for concordance and unique findings. Rearrangements were classified as typical or atypical (e.g., noncanonical partners or cryptic breakpoints). Sensitivity was evaluated based on the detection of clinically significant variants. Results from both assays were compared for their clinical relevance. Statistical analysis included concordance rates and detection thresholds to evaluate the Hi-C assay’s performance against FISH. The study also assessed the assay’s ability to detect novel or cryptic alterations in a hypothesis-free manner, ensuring comprehensive genomic profiling.

### 2.5. Immunohistochemistry Assay

Immunohistochemistry (IHC) for the MYC, BCL2, and BCL6 protein was performed on the FFPE specimen using their monoclonal antibody 9E10, 100/D5, and BCL-DWN, respectively (Catalog #MA1-980, #MA5-11757, #17-5453-82, ThermoFisher Scientific, Waltham, MA, USA) according to the manufacturer’s protocol.

## 3. Results

The Hi-C-based lymphoma assay consistently outperformed FISH in detecting gene rearrangements and fusions across all five cases, identifying actionable variants missed by conventional FISH methods. Key findings are summarized below and in Table 1. Atypical rearrangements detected by FISH in this study included three cases with *MYC* rearrangement (two also included amplification), three cases with *BCL6* rearrangement, and one case with an *IGH*::*MYC* fusion.

### 3.1. Case-Specific Findings

#### 3.1.1. Case #1

**Figure 1:**
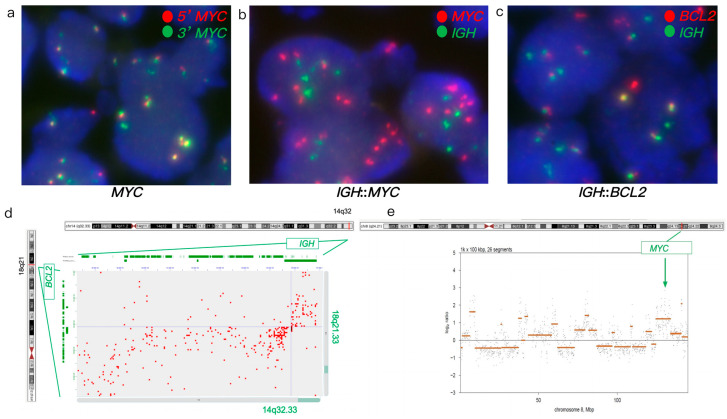
FISH and Hi-C data in case 1. (**a**–**c**) FISH on FFPE tumor specimen. (**a**) MYC break-apart FISH showed atypical MYC, amplified MYC (yellow signals) along with additional one to two green signals. (**b**) Dual-fusion *IGH*::*MYC* showed gain/amplification of MYC (multiple red signals) without *IGH*::*MYC* fusion. (**c**) Dual-fusion *IGH*::*BCL2* probes showed *IGH*::*BCL2* fusion (yellow signals). (**d**,**e**) Hi-C data on FFPE tumor specimen. (**d**) Hi-C data revealed *IGH*::*BCL2* fusion. (**e**) Hi-C data revealed a complex abnormal chromosome 8 with gain and amplification of MYC (the orange lines representing the log2 ratio of this region, was significantly above the baseline).

FISH analysis of a DLBCL tumor revealed an atypical *MYC* rearrangement with gain and amplification (66% of nuclei, including 34% with a 3–5+ fusion signal plus one green signal (3-ampF1G) and 32% with 3-ampF2G), a *BCL2* rearrangement (73% of nuclei), and an *IGH*::*BCL2* fusion (76% of nuclei) (Figure 1a–c). IHC analysis showed positive expression of BCL2 and BCL6, with negative expression of MYC. Hi-C confirmed *IGH*::*BCL2* fusion and identified *MYC* gain/amplification along with additional chromosome 8 rearrangements, indicating complex structural variants (Figure 1d,e). Furthermore, Hi-C also revealed additional rearrangements involving *KDSR*, *TNFRSF11A*, *BCR*, *LGL*, *LYN*, *PLAG1*, and *DENND3* genes. Hi-C analysis revealing *MYC* amplification concurrent with an *IGH*::*BCL2* fusion supported a diagnosis of DLBCL, distinct from HGBCL with concurrent *MYC* and *BCL2* rearrangements. This molecular profile, characterized by *MYC* amplification rather than rearrangement in the presence of an *IGH*::*BCL2* fusion, suggests that a tailored treatment regimen, distinct from that used for HGBCL with concurrent *MYC* and *BCL2* rearrangements, may be necessary to optimize clinical outcomes in DLBCL [32,33,34,35,36].

#### 3.1.2. Case #2

**Figure 2:**
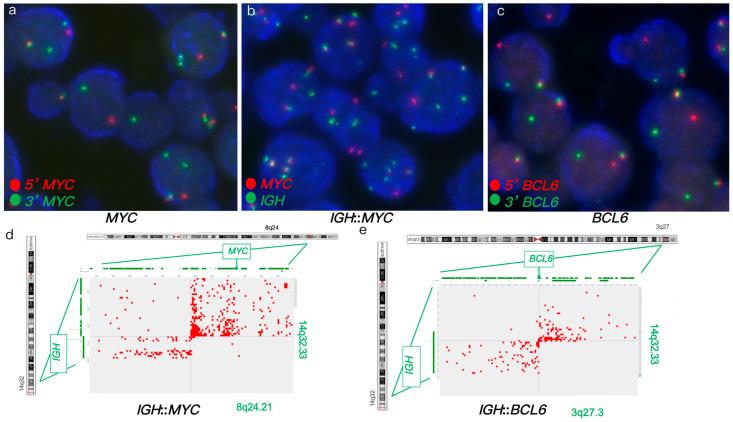
FISH and Hi-C data in case 2. (**a**,**b**) FISH on FFPE tumor specimen. (**a**) *MYC* break-apart FISH showed atypical *MYC* rearrangements. (**b**) Dual-fusion *IGH*::*MYC* FISH showed *IGH*::*MYC* fusions (yellow signals). (**c**) *BCL6* break-apart FISH showed atypical *BCL6* rearrangement. (**d**,**e**) Hi-C data on FFPE tumor specimen. (**d**) Hi-C data revealed *IGH*::*MYC* fusion. (**e**) Hi-C data revealed *IGH*::*BCL6* fusion.

FISH analysis of a DLBCL tumor identified an atypical *MYC* rearrangement in 84% of nuclei, showing atypical patterns (1R2G1F) (Figure 2a). It also detected *IGH*::*MYC* fusion in 96% of nuclei, with atypical patterns (1R2G2-3F, 2R2G2F), as well as *BCL6* rearrangement in 75% of nuclei (Figure 2b,c). IHC analysis showed positive expression of BCL6 and MYC. Hi-C analysis confirmed the *IGH*::*MYC* fusion and *BCL6* rearrangement, further identifying *IGH* as the translocation partner in the *BCL6* rearrangement, forming an *IGH*::*BCL6* fusion (Figure 2d,e). In this case, *IGH* was implicated in both *MYC* and *BCL6* rearrangements. Furthermore, Hi-C revealed additional rearrangements involving *POU2AF1*, *E2F2*, *ID3*, and *MDS2* genes. Hi-C analysis of *IGH*::*MYC* and *IGH*::*BCL6* fusions supported a HGBCL diagnosis, likely to benefit from intensified regimens to optimize treatment outcomes.

#### 3.1.3. Case #3

**Figure 3:**
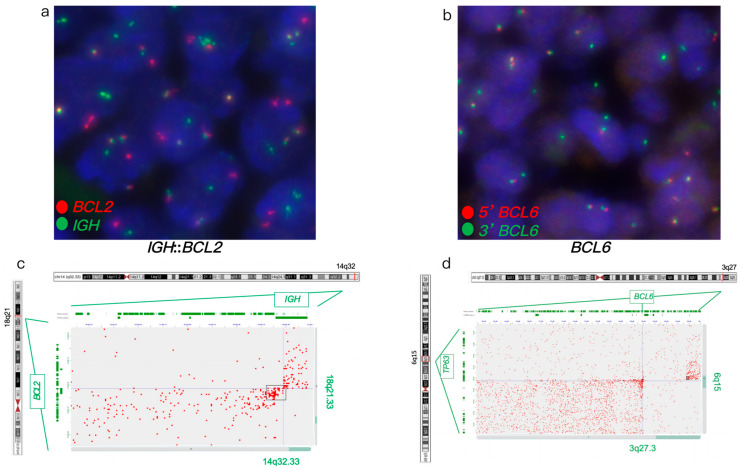
FISH and Hi-C analysis in case 3. (**a**,**b**) FISH analysis of FFPE tumor specimen. (**a**) Dual-fusion *IGH*::*BCL2* probe revealing *IGH*::*BCL2* fusion (yellow signals). (**b**) *BCL6* break-apart FISH showing an atypical *BCL6* rearrangement with a 1G1F signal pattern and loss of the 5’ *BCL6* region (red signal). (**c**,**d**) Hi-C analysis of FFPE tumor specimen. (**c**) Hi-C data showing *IGH*::*BCL2* fusion. (**d**) Hi-C data identifying an atypical *BCL6* rearrangement with a 7 Mb deletion 5’ of *BCL6*. Mb: megabases.

FISH analysis of a large B-cell lymphoma revealed an atypical *BCL6* rearrangement with a 1G1F signal pattern and an *IGH*::*BCL2* fusion (Figure 3a,b). IHC analysis showed positive expression of BCL2 and BCL6. Hi-C analysis confirmed these FISH findings and further characterized the atypical *BCL6* rearrangement with a 7 Mb deletion of the 5’ *BCL6* region, encompassing the *TP63* gene (Figure 3c,d). Furthermore, Hi-C identified rearrangements involving the *TNFRSF11A*, *KDSR*, *CLTC*, *HLF*, *LYN*, *TFRC*, *KAT6A*, *PLAG1*, *PRKCA*, and *NDRG1* genes, as well as loss of *RAB29* and *MSI2*. Hi-C analysis identified gene rearrangements and copy number variants in this case, but their lack of established diagnostic and clinical significance means they may not currently influence diagnosis or treatment decisions.

#### 3.1.4. Case #4

**Figure 4:**
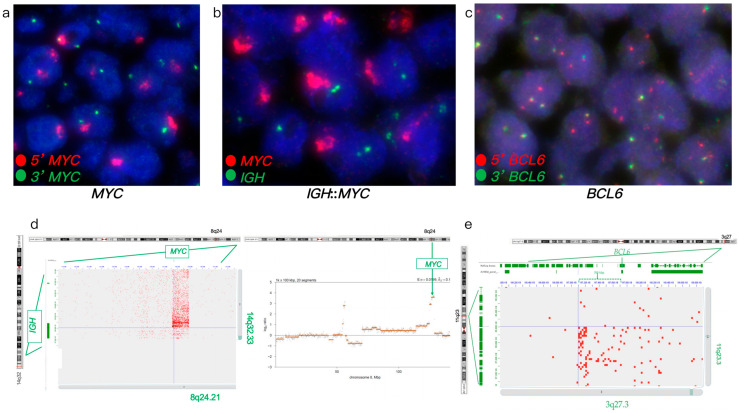
FISH and Hi-C analysis in case 4. (**a**–**c**) FISH analysis of FFPE tumor specimen. (**a**) *MYC* break-apart FISH showing atypical *MYC* rearrangements with amplification of the 5’ *MYC* region (red signals) and split red and green signals (ampR1-3G). (**b**) Dual-fusion *IGH*::*MYC* showing *MYC* amplification (multiple red signals) without *IGH*::*MYC* fusion. (**c**) *BCL6* break-apart FISH identifying atypical *BCL6* rearrangements (1-3R2-3F signal pattern). (**d**–**e**) Hi-C analysis of FFPE tumor specimen. (**d**) Hi-C data confirming *MYC* amplification and identifying an atypical *IGH*::*MYC* fusion resulting from *MYC* insertion. (**e**) Hi-C data revealing an atypical *BCL6* rearrangement resulting from atypical t(3;11) translocation.

FISH analysis of a large B-cell lymphoma revealed an atypical *MYC* rearrangement with amplification (ampR1-3G), an atypical *BCL6* rearrangement (1-3R2-3F signal pattern), and no *IGH*::*MYC* fusion (Figure 4a–c). Hi-C analysis confirmed the atypical *MYC* rearrangement with amplification and identified an atypical *IGH*::*MYC* fusion resulting from an insertional *MYC* event into *IGH*, typically cryptic by FISH (Figure 4d). Hi-C further revealed that the atypical *BCL6* rearrangement resulted from an atypical t(3;11) translocation (Figure 4d). Additionally, Hi-C detected *TP53* loss, *KMT2A* amplification with t(8;11), gains/amplifications of *PLAG1* and *LYN*, rearrangements involving *ERBB2*, *FOXO1*, *S1PR2*, *TYK2*, *NF1*, *PYCR1*, *FSTL3*, *LPP*, *PRDM16*, *KMT2A*, *LYN*, and *PLAG1* genes, and complex rearrangement of chromosome 3. *TP53* loss and *KMT2A* amplification were further confirmed by follow-up FISH testing. Hi-C analysis revealing *IGH*::*MYC*, *TP53* loss, *KMT2A* alterations, and a complex genome supported a high-risk DLBCL diagnosis, likely to benefit from targeted clinical trials to optimize treatment outcomes.

#### 3.1.5. Case #5

**Figure 5:**
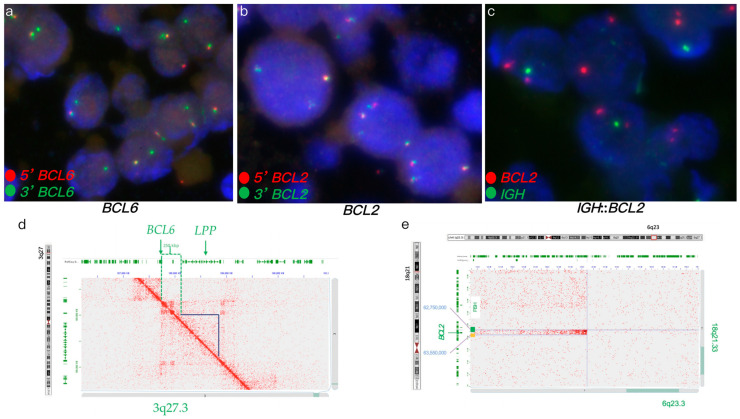
FISH and Hi-C analysis in case 5. (**a**–**c**) FISH analysis of FFPE tumor specimen. (**a**) *BCL6* break-apart FISH revealing an atypical *BCL6* rearrangement with a 1G1F signal pattern. (**b**) *BCL2* break-apart FISH indicating no rearrangement (normal pattern). (**c**) Dual-fusion *IGH*::*BCL2* FISH showing no *IGH*::*BCL2* fusion (normal pattern). (**d**,**e**) Hi-C analysis of FFPE tumor specimen. (**d**) Hi-C data confirming the atypical *BCL6* rearrangement with a deletion of the *LPP* gene. (**e**) Hi-C data identifying an atypical *BCL2* rearrangement resulting from an insertional t(6;18) translocation. Green and yellow bars represent break-apart *BCL2* FISH probes. Chromosomal review of Hi-C data is shown in Supplementary Figure S1.

FISH analysis of an aggressive B-cell lymphoma revealed an atypical *BCL6* rearrangement with a 1G1F signal pattern, no *BCL2* rearrangements, and no *IGH*::*BCL2* fusion (Figure 5a–c). IHC analysis showed positive expression of BCL2 and BCL6. Hi-C analysis confirmed the atypical *BCL6* rearrangement with a deletion of the *LPP* gene (Figure 5d). Additionally, Hi-C identified atypical *BCL2* rearrangements involving insertional t(6;18) and t(4;18) translocations, typically cryptic by FISH (Figure 5e). Hi-C further detected losses of *CDKN2A*, *CDKN2B*, *TNFAIP3*, *BCL7A*, and *PCM1* genes, gain of 18q, and novel rearrangements involving *FGFR1*, *FGFR2*, *TP63*, *KDSR*, *TET1*, *LYN*, and *NSD3* genes. Hi-C analysis revealed *BCL2* rearrangements, losses of *CDKN2A*, *CDKN2B*, and *TNFAIP3*, and a complex genome profile, supporting a high-risk DLBCL diagnosis and suggesting potential benefit from targeted clinical trials to enhance treatment outcomes.

### 3.2. Comparative Performance

The Hi-C-based lymphoma assay outperformed FISH across all five DLBCL cases, detecting clinically significant gene fusions and rearrangements missed by FISH (Table 1). FISH identified atypical *MYC* rearrangements (Cases 1, 2, 4), *BCL6* rearrangements (Cases 2, 3, 4, 5), *BCL2* rearrangements (Case 1), and *IGH*::*MYC* or *IGH*::*BCL2* fusions (Cases 1, 2, 3). However, its reliance on predefined probes limited detection of cryptic events, such as insertional *IGH*::*MYC* fusion (Case 4) and *BCL2* rearrangements via t(6;18) and t(4;18) (Case 5) (Supplemental Table S3).

Hi-C confirmed all FISH-detected rearrangements with 100% concordance and identified additional actionable variants, including noncanonical *IGH*::*BCL6* fusion (Case 2), a 7 Mb deletion 5’ of *BCL6* encompassing *TP63* (Case 3), *TP53* loss, *KMT2A* amplification with t(8;11), and complex rearrangement of chromosome 3 (Case 4). It also detected novel rearrangements involving genes such as *POU2AF1, E2F2, ID3, MDS2* (Case 2), *TNFRSF11A*, *KDSR*, *CLTC*, *HLF*, *LYN*, *TFRC*, *KAT6A*, *PLAG1*, *PRKCA*, *NDRG1* (Cases 1, 3, 4), *ERBB2*, *FOXO1*, *S1PR2*, *TYK2*, *NF1*, *PYCR1*, *FSTL3*, *PRDM16* (Case 4), and *FGFR1*, *FGFR2*, *TET1*, *NSD3* (Case 5), alongside losses of *RAB29*, *MSI2* (Case 3), *CDKN2A*, *CDKN2B*, *TNFAIP3*, *BCL7A*, *PCM1*, and gain of 18q (Case 5). Specificity was maintained, as Hi-C accurately reported no rearrangements in regions where FISH indicated normal patterns, except for insertional rearrangements (e.g., *BCL2* in Case 5).

Hi-C’s whole-genome profiling reduced the need for sequential FISH probing, minimizing tissue consumption and turnaround time. These results highlight its potential as a superior diagnostic tool for complex lymphoma cases.

## 4. Discussion

The Hi-C-based lymphoma assay represents a significant advancement in lymphoma diagnostics, addressing critical limitations of FISH by providing a high-resolution, whole-genome approach to detecting gene fusions and rearrangements in DLBCL. This study demonstrates that the Hi-C assay outperforms conventional FISH, identifying actionable genetic alterations. The actionable findings included cryptic breakpoints, noncanonical fusion partners, and complex structural variants across five atypical lymphoma cases. These findings have profound implications for diagnostic accuracy, subtype classification, prognostic assessment, and personalized treatment strategies.

FISH, while a cornerstone of lymphoma diagnostics, is constrained by its reliance on predefined probes and limited genomic coverage, which can miss atypical or complex rearrangements. In this study, FISH identified *MYC* rearrangements in Cases 1, 2, and 4, *BCL6* rearrangements in Cases 2, 3, 4, and 5, *BCL2* rearrangements in Case 1, and *IGH*::*MYC* or *IGH*::*BCL2* fusions in Cases 1, 2, and 3 (Section 3.1). However, it failed to detect critical events such as the insertional *IGH*::*MYC* fusion in Case 4, insertional *BCL2* rearrangements t(6;18) and t(4;18)) in Case 5, and noncanonical *IGH*::*BCL6* fusion in Case 2. These limitations underscore FISH’s challenges in detecting cryptic or noncanonical alterations, which are increasingly recognized as clinically significant in DLBCL and HGBCL per WHO 2016 and 2022 classifications.

While *MYC* translocations have been extensively studied in DLBCL, the prognostic significance of *MYC* gain or amplification remains controversial [32,33,34,35,36]. Earlier studies suggested that *MYC* gain or amplification may be associated with aggressive disease and poor prognosis [32,33,34]. However, recent publications indicate that these alterations may not correlate with overall survival or hold prognostic significance [35,36]. Distinguishing between *MYC* translocations and *MYC* gain or amplification is critical in DLBCL due to their distinct clinical implications. However, detecting concurrent low-level *MYC* translocations using *MYC* break-apart FISH probes on FFPE DLBCL samples is challenging, particularly when multiple *MYC* gene copies are present in the nucleus. In such cases, the Hi-C assay is a valuable tool for differentiating between *MYC* amplification with and without *MYC* rearrangement, as demonstrated in cases 1 and 4 of this study.

The Hi-C assay’s diagnosis-agnostic approach, assessing several hundred lymphoma-associated genes in a single test, reduces the need for sequential FISH probing, thereby minimizing tissue consumption and turnaround time. This is particularly advantageous for FFPE samples, where tissue availability is often limited. Our approach leverages the enrichment of all ligated Hi-C sequence pairs across the genome making this a whole genome sequencing (WGS) approach to capture the entire genome, identifying structural and sequence variations without selecting specific regions upfront. We then apply targeted analysis to study lymphoma-associated loci (417 genes/regions) and their interaction partners, combining broad coverage with specific focus. This genome-wide approach allows discovery of unexpected variants while focusing on regions of clinical importance reduces expedites the analysis time. In contrast, target-capture Hi-C enriches specific genomic regions, like a gene panel, using capture probes to reduce sequencing costs. Target-capture is useful for studies targeting known regions but may miss broader genomic insights. Therefore, our WGS with targeted analysis offers a more exploratory and comprehensive approach compared to targeted-capture Hi-C, enabling broader genomic discovery while maintaining focused analysis of key regions.

This study demonstrated Hi-C’s ability to delineate *MYC*, *BCL6*, and *BCL2* rearrangements amid complex genomes, including non-IG partners or additional losses/gains not discernible by FISH. These refinements stem from Hi-C’s topological data, which reveals A/B compartment shifts, topologically associating domain disruptions, and neo-loop formations indicative of enhancer hijacking—mechanisms where oncogenes like *MYC* or *BCL2* are aberrantly activated by ectopic enhancers. Such details distinguish functional (e.g., promoter-swap) from non-functional rearrangements, reducing false positives and enabling accurate subtyping, as seen in Hi-C’s detection of *IGH*::*IRF4* in pediatric large B-cell lymphoma cases missed by FISH and NGS [23].

In contrast, the Hi-C assay’s whole-genome profiling enabled the detection of all FISH-identified rearrangements with full concordance while uncovering additional actionable variants. For instance, Hi-C confirmed *IGH*::*BCL2* and *IGH*::*MYC* fusions and revealed novel rearrangements involving genes such as *POU2AF1*, *E2F2*, *ID3*, *MDS2* (Case 2), *TNFRSF11A*, *KDSR*, *CLTC*, *HLF*, *LYN*, *TFRC*, *KAT6A*, *PLAG1*, *PRKCA*, *NDRG1* (Cases 1, 3, 4), *ERBB2*, *FOXO1*, *S1PR2*, *TYK2*, *NF1*, *PYCR1*, *FSTL3*, *PRDM16* (Case 4), and *FGFR1*, *FGFR2*, *TET1*, *NSD3* (Case 5). It also identified significant structural variants, including *TP53* loss and complex rearrangement of chromosome 3 (Case 4), a 7 Mb deletion 5’ of *BCL6* encompassing *TP63* (Case 3), and losses of *CDKN2A*, *CDKN2B*, *TNFAIP3*, *BCL7A*, *PCM1*, *RAB29*, *MSI2*, and gain of 18q (Case 5). The detection of complex rearrangement events and large deletions (e.g., 7 Mb deletion in Case 3) underscores Hi-C’s capability to characterize complex structural variants that may drive aggressive disease phenotypes. These findings highlight Hi-C’s ability to detect complex genomic events that influence prognosis and therapeutic decision-making, such as *TP53* loss, which is associated with poor outcomes in DLBCL, and *KMT2A* amplification, which may indicate eligibility for targeted therapies.

Hi-C analysis offers prognostic precision by elucidating genomic complexities that stratify DLBCL/HGBCL patients into risk groups, guiding survival predictions and therapeutic strategies. Genetic subtypes profoundly influence prognosis, with *MYC*/*BCL2* rearrangements defining highly aggressive entities associated with reduced median overall survival (OS) under standard treatment strategies. Hi-C enhances risk stratification by accurately detecting these rearrangements and co-occurring alterations, such as *TP53* losses or complex copy number variants, which exacerbate poor outcomes, as observed in high-risk cases (e.g., Case 4: *TP53* loss; Case 5: complex copy number variants). Notably, Hi-C-identified *IGH*::*MYC* rearrangements (e.g., Cases 2, 4), driven by potent immunoglobulin enhancers, correlate with inferior OS compared to non-IGH partners due to enhanced oncogene expression [37]. Topological analyses further refine prognostication by quantifying enhancer hijacking; neo-loops linking *MYC* to super-enhancers predict aggressive biology, particularly in cases with *MYC* amplification and low immune infiltration (e.g., Cases 1, 4). In the cases described, Hi-C’s detection of complex genomes aligned with high-risk features, prompting referrals to clinical trials targeting specific molecular drivers. For instance, Case 5’s complex Hi-C profile supports enrollment in trials targeting *BCL2* rearrangements (e.g., venetoclax, selinexor), NF-κB activation from *TNFAIP3* loss (e.g., lenalidomide, ibrutinib), or cell cycle dysregulation from *CDKN2A*/*CDKN2B* deletions (e.g., CDK4/6 inhibitors like palbociclib). Novel rearrangements involving *FGFR1*, *FGFR2*, *TP63*, *KDSR*, *TET1*, *LYN*, or *NSD3* remain less targeted due to their rarity, but trials such as pemigatinib (NCT02924376), acalabrutinib (NCT04094142), or belinostat (NCT01839097) may address related pathways like FGFR signaling, BCR/NF-κB, or epigenetic dysregulation. Additionally, immunotherapy trials, including CAR T-cell (NCT03391466 or NCT04231747) therapies, offer broad applicability for complex genetic profiles, enhancing treatment options for high-risk DLBCL/HGBCL.

Despite its advantages, the Hi-C assay has limitations. The requirement for high-throughput sequencing and specialized bioinformatics pipelines may pose implementation challenges in resource-limited settings. While Hi-C’s whole-genome coverage provides comprehensive profiling, the clinical significance of some novel rearrangements (e.g., *POU2AF1*, *E2F2*) remains to be fully elucidated, necessitating further functional studies. For instance, this study identified recurrent *PLAG1* and *LYN* rearrangements in four of five DLBCL/HGBCL cases suggests their potential as novel biomarkers, warranting further investigation into their oncogenic roles and therapeutic implications. The cost of Hi-C sequencing and data analysis may also be a barrier to widespread adoption, although its ability to replace multiple FISH tests could offset expenses in the long term. Additionally, prognostic applications face challenges: Hi-C’s sensitivity to subclonal events may inflate perceived genomic complexity, necessitating integration with clinical scores (e.g., the international prognostic index) for comprehensive risk assessment. Future prospective studies should validate Hi-C-derived signatures to establish standardized cutoffs for identifying high-risk features in DLBCL/HGBCL. Furthermore, due to limited specimen availability, we could not perform short-read WGS in parallel to provide complementary data for validating and contextualizing the Hi-C findings in this study. While this proof-of-concept study highlights Hi-C’s diagnostic potential for lymphoma, larger cohort studies of diverse lymphoma cases, utilizing both FISH and Hi-C, are needed to ensure robust statistical analysis and validate Hi-C’s clinical utility.

Overall, the clinical implications of these findings are significant. The Hi-C assay’s ability to detect cryptic and noncanonical alterations supports precise DLBCL subtype classification (e.g., germinal center B-cell-like versus activated B-cell-like) and identifies high-risk features, such as double-hit signatures, that guide risk stratification and treatment decisions. For example, the identification of *IGH*::*MYC* and *IGH*::*BCL2* fusions in Cases 1, 2, and 4 aligns with high-grade DLBCL, which may benefit from intensified regimens or clinical trials targeting *MYC* or *BCL2*. The detection of novel gene rearrangements and genomic alterations involving targetable genes suggests potential for precision therapies, enhancing personalized treatment strategies.

Future studies should focus on validating the Hi-C assay in larger cohorts to establish its diagnostic and prognostic utility across diverse lymphoma subtypes. Additionally, functional studies of novel biomarkers, such as *PLAG1* and *LYN*, are needed to clarify their roles in lymphomagenesis and therapeutic response. Optimization of the assay for FFPE samples, including streamlining bioinformatics pipelines, will further enhance its clinical feasibility. Comparative cost-effectiveness analyses against FISH and other genomic assays (e.g., whole-genome sequencing) will also be critical to support broader adoption.

## 5. Conclusions

The Hi-C-based lymphoma assay offers a transformative diagnostic tool that overcomes FISH’s limitations, providing high-resolution, comprehensive detection of gene fusions and rearrangements. Its ability to identify actionable and novel genomic alterations supports its potential as a complementary or superior approach to FISH, paving the way for improved diagnostic accuracy and personalized therapeutic strategies in lymphoma management.

## Figures and Tables

**Table 1:** Summary of FISH AND Hi-C lymphoma assay results in this study.

Case #	FISH Results					Hi-C Lymphoma Assay Results						
	*MYC*-R.	*IGH*::*MYC* Fusion	*BCL2*-R.	*IGH*::*BCL2* Fusion	*BCL6*-R.	*MYC*-R.	*IGH*::*MYC* Fusion	*BCL2*-R.	*IGH*::*BCL2* Fusion	*BCL6*-R.	Additional Gene Rearrangement/Fusions	Additional Copy Number Variants
1	Atypical with gain/amp. (ampF1-2G)	-	+	+	-	Gain/amp.	-	+	+	-	*KDSR*, *TNFRSF11A*, *BCR*, *LYN*, *PLAG1*, and *DENND3*	
2	Atypical (1R2G1F)	Atypical (1R2G1-3F, 2R2G2F)	-	-	+	+	+	-	-	*IGH*::*BCL6*	*POU2AF1*, *E2F2*, *ID3*, and *MDS2*	
3	-	-	+	+	Atypical (1G1F)	-	-	+	+	Atypical with a 7 Mb deletion 5′ of *BCL6*	*TNFRSF11A*, *KDSR*, *CLTC*, *HLF*, *LYN*, *TFRC*, *KAT6A*, *PLAG1*, and *NDRG1*	Loss of *RAB29* and *MSI2*
4	Atypical with amp. (ampR1-3G)	-	-	-	Atypical (1-3R2-3F)	Atypical with amp. (7–9 copies)	+	-	-	Atypical t(3;11) ~700 Kb from breakpoint	*ERBB2*, *FOXO1*, *S1PR2*, *TYK2*, *NF1*, *PYCR1*, *FSTL3*, *LPP*, *PRDM16*, *KMT2A*, *LYN*, *PLAG1*, *IGH*::*MIR4507* fusion	Loss of *TP53*, amp. of *KMT2A* and *PLAG1*, gain of *LYN*, complex rearrangement of Chromosome 3
5	-	-	-	-	Atypical (1G1F)	-	-	+: t(6;18) insertion and t(4;18)	-	Atypical with deletion of LPP	*FGFR1*, *FGFR2*, *TP63*, *KDSR*, *TET1*, *LYN*, and *NSD3*	losses of *CDNK2A*/*2B*, *TNFAIP3*, *BCL7A*, and *PCM1*, gain of 18q

amp.: amplification; Kb: kilo base pair; Mb: megabases; R.: Rearrangement; “-”: negative; “+”: positive.

## Data Availability

The dataset used for the current study is available from the corresponding author upon reasonable request.

## Supplementary Materials

The following supporting information can be downloaded at: https://www.mdpi.com/article/10.3390/genes16091093/s1, Supplementary Table S1: Sample and Hi-C experimental specifications. Supplementary Table S2: 417 lymphoma-associated genes. Supplementary Table S3: Potential diagnostic and therapeutic implications of Hi-C findings. Supplementary Figure S1: Chromosomal view of Hi-C data.

## Abbreviations

The following abbreviations are used in this manuscript:

DLBCL	Diffuse large B-cell lymphoma
FISH	Fluorescence in situ hybridization
FFPE	Formalin-fixed paraffin-embedded
HGBCL	High grade B-cell lymphoma
NHL	Non-Hodgkin lymphoma
WGS	Whole genome sequencing
