# Supplementary material for: Hi-C Technology Reveals Actionable Gene Fusions and Rearrangements in Diffuse Large B-Cell Lymphoma Unidentified by Conventional FISH
Source: Genes (Basel). 2025 Sep 16;16(9):1093. doi: 10.3390/genes16091093 (PMC12469463; doi:10.3390/genes16091093)

Case 1: chromosomal view

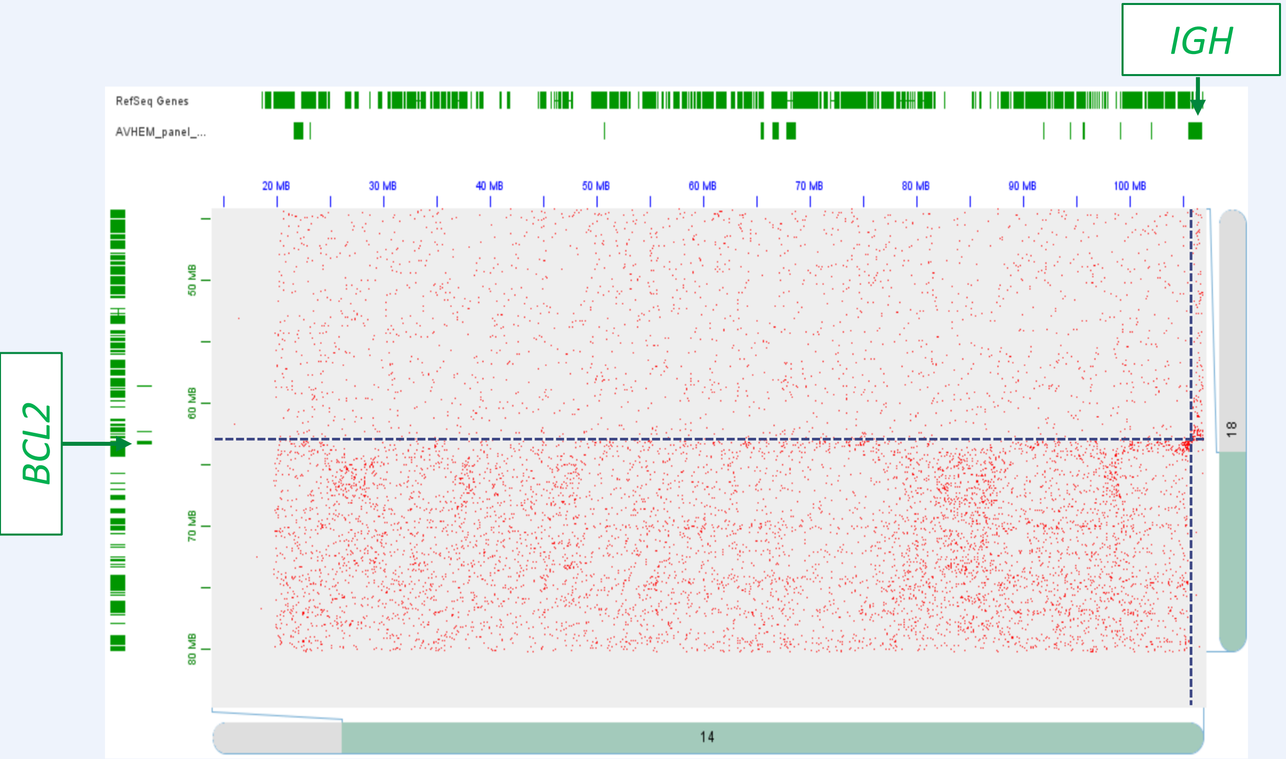

Case 2: : chromosomal view

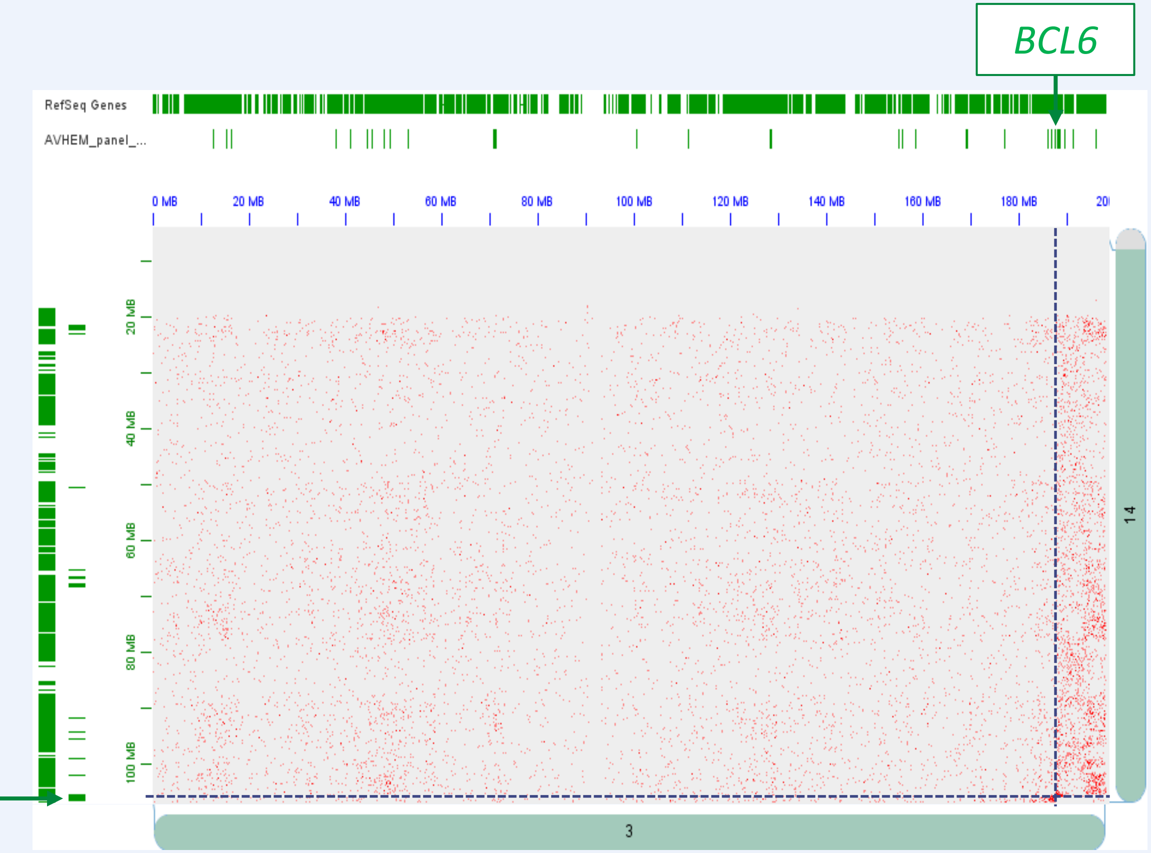

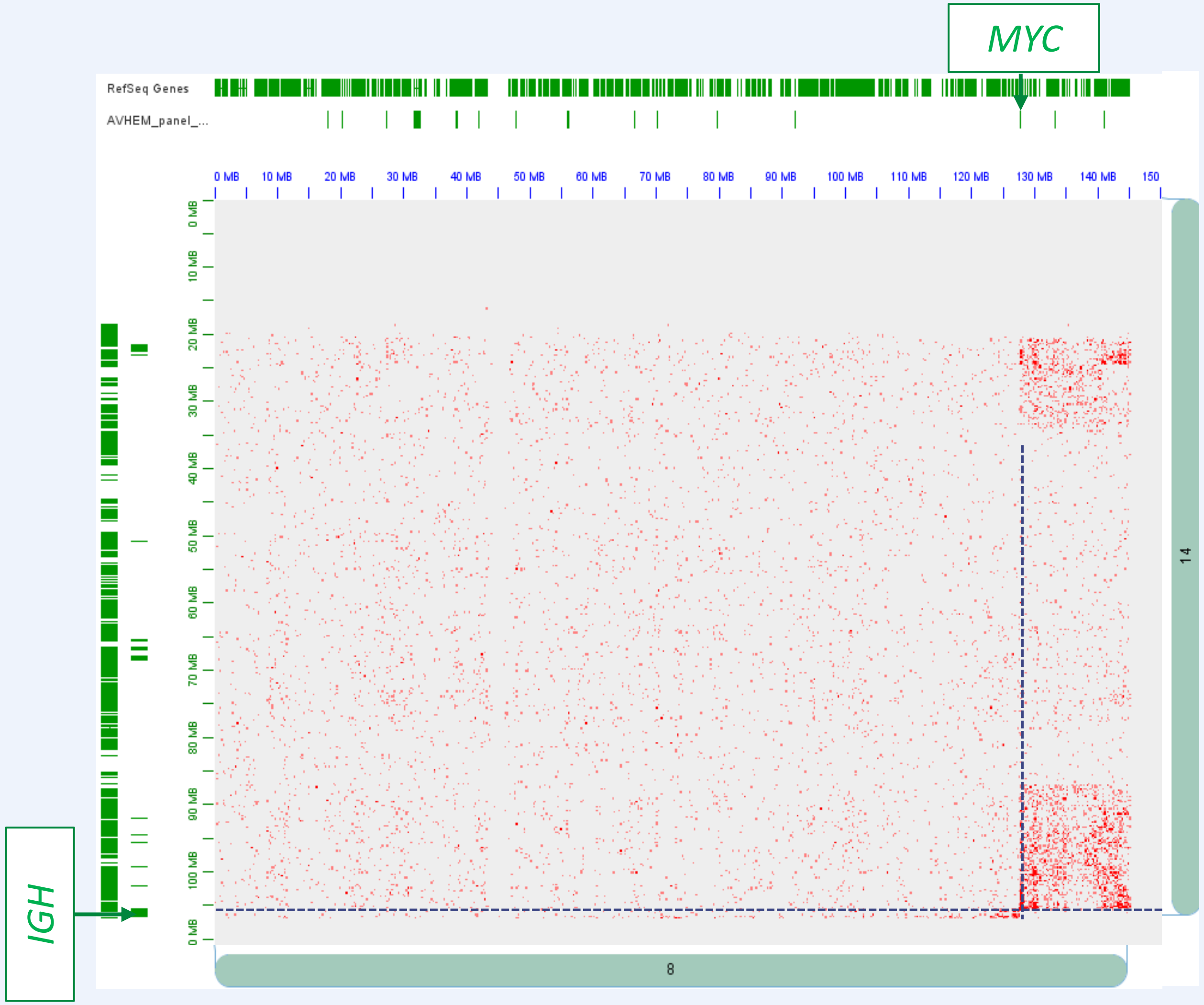

Case 3: : chromosomal view

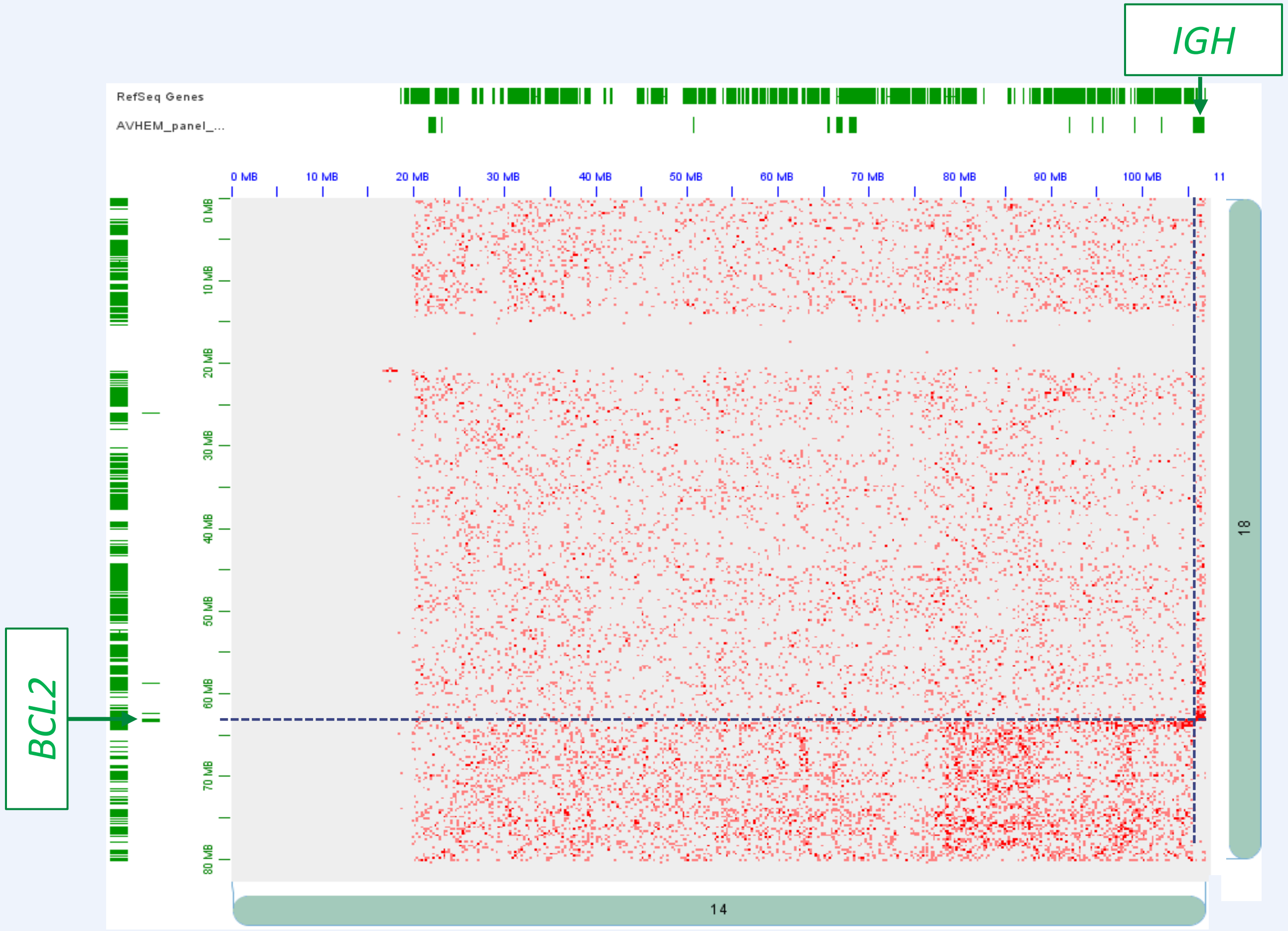

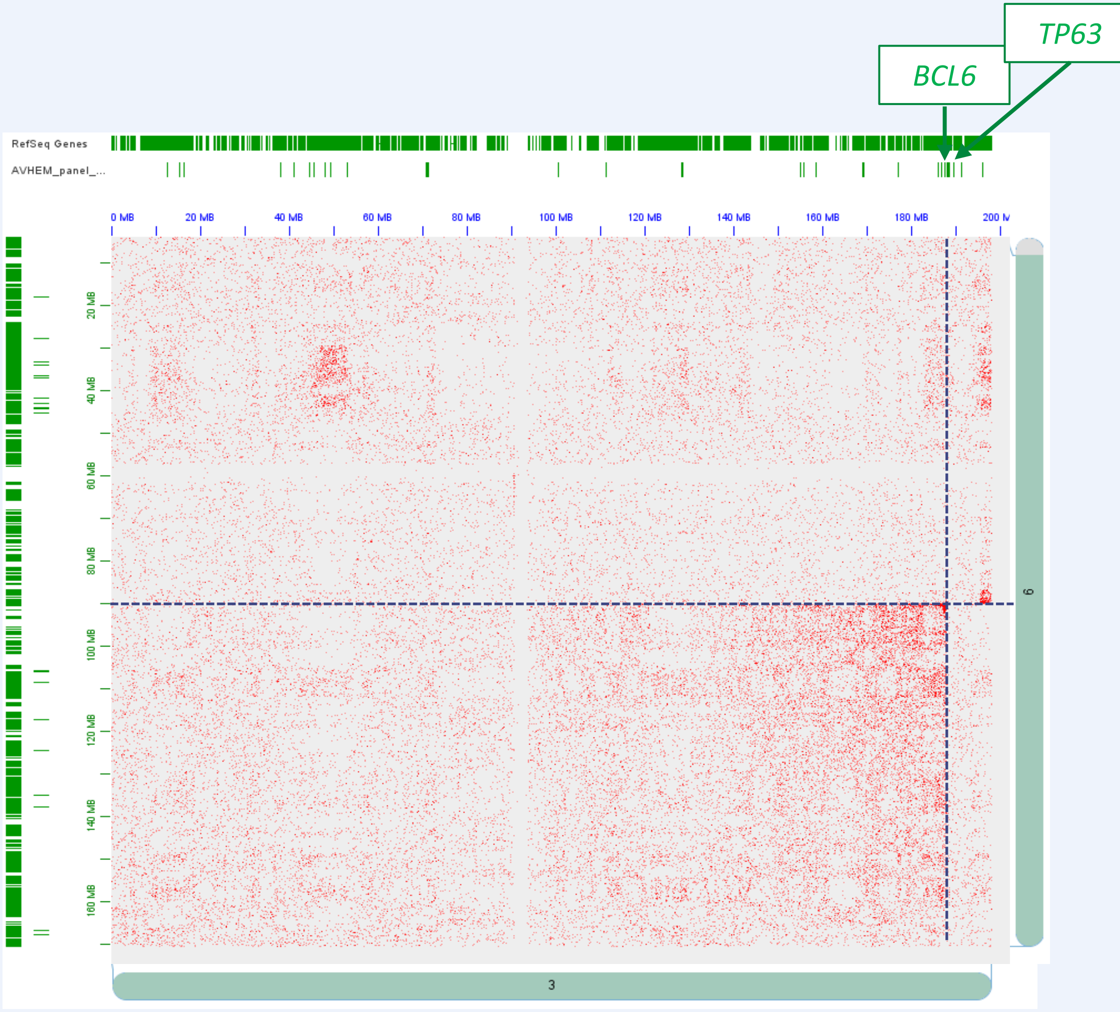

Case 4: : chromosomal view

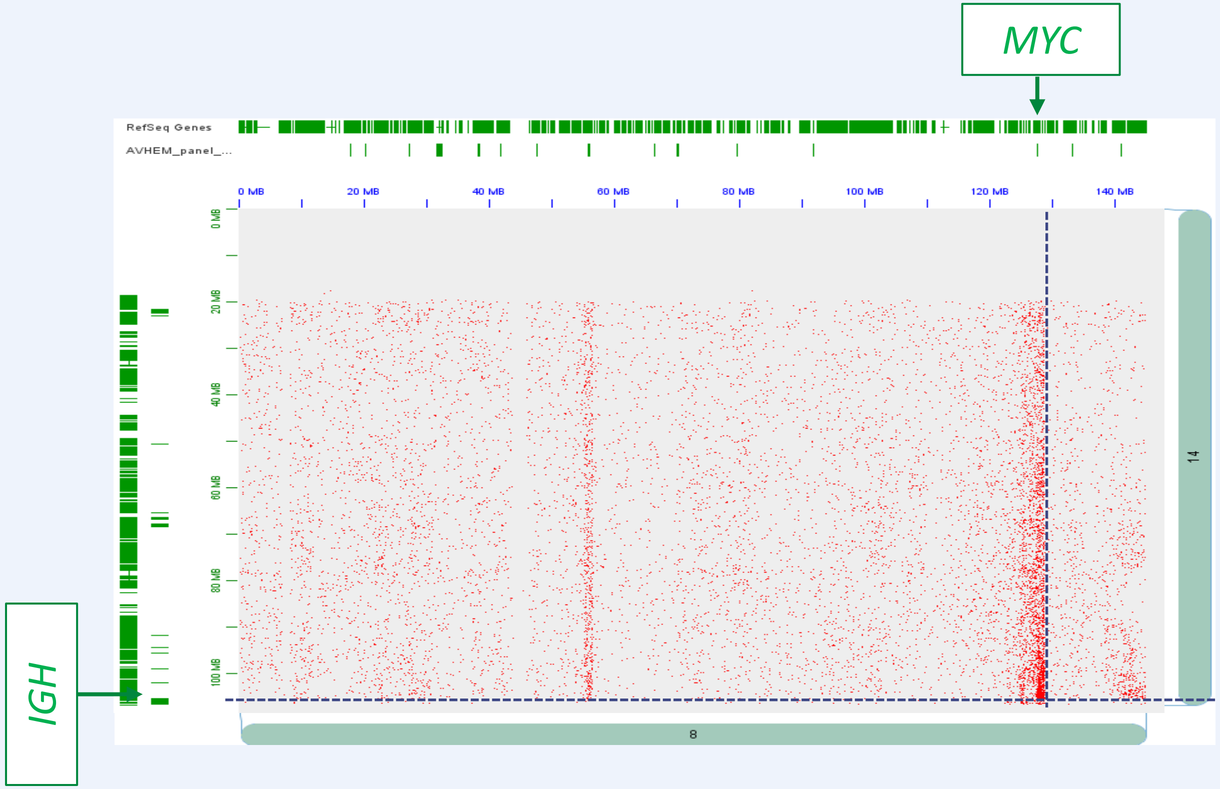

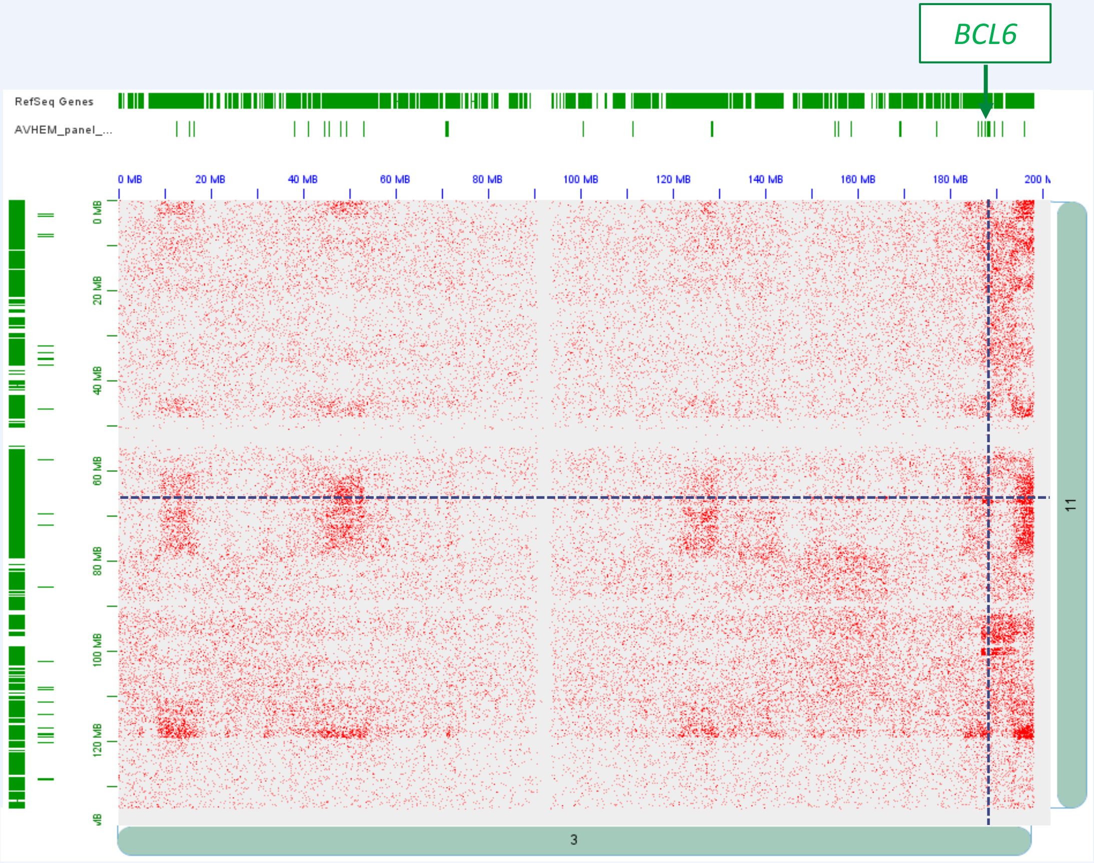

Case 5: chromosomal view

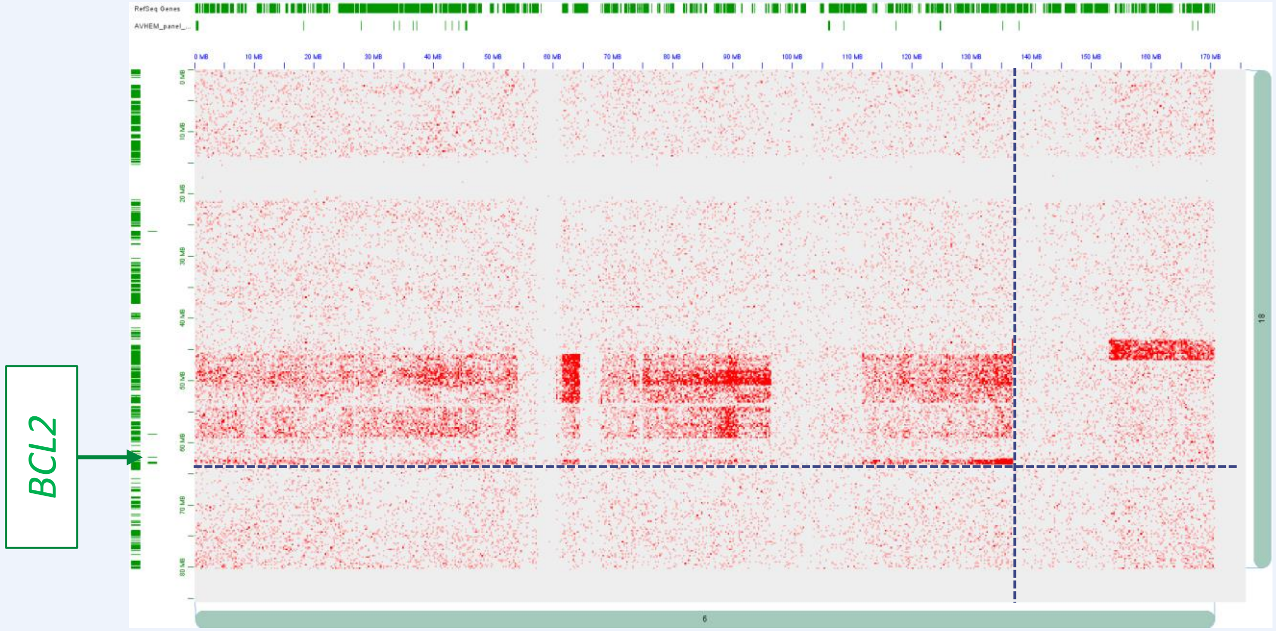

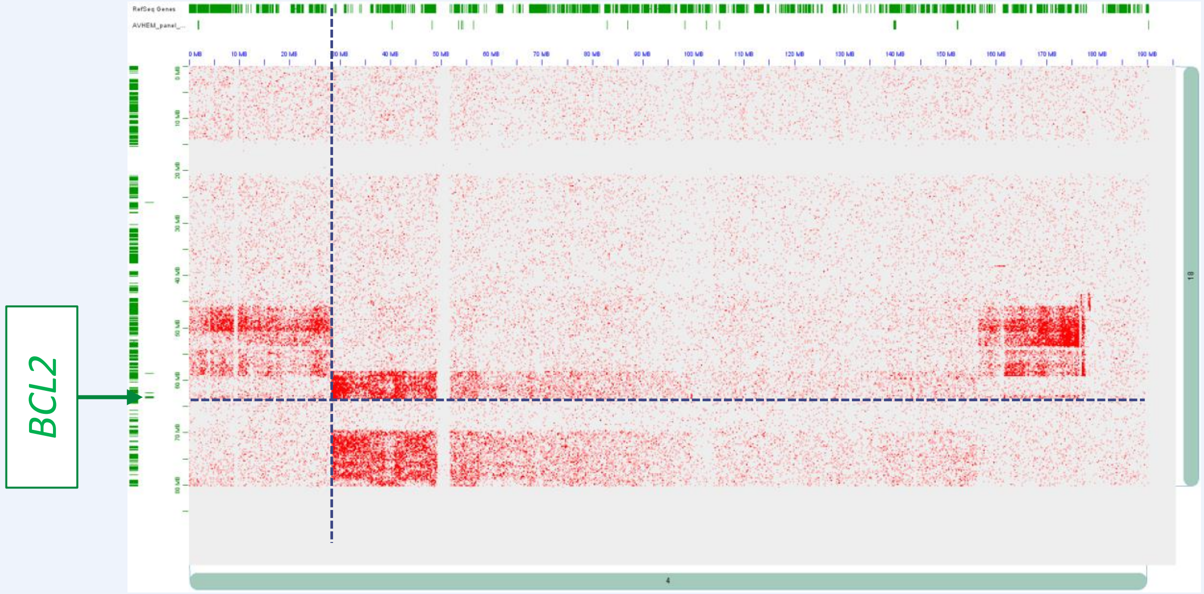

Supplement: Supplementary file 1 [file genes-16-01093-s001.zip › genes-3820818-Supplemental Figure S1.pdf]
