# Supplementary material for: Hi-C Technology Reveals Actionable Gene Fusions and Rearrangements in Diffuse Large B-Cell Lymphoma Unidentified by Conventional FISH
Source: Genes (Basel). 2025 Sep 16;16(9):1093. doi: 10.3390/genes16091093 (PMC12469463; doi:10.3390/genes16091093)
Supplement: Supplementary file 1 [file genes-16-01093-s001.zip › Supplemental Table S2.pdf]

Supplemental Table S2: 417 lymphoma-associated genes

ABI1	*BCL3*	*CD79B*	*CSF1R*	*EPOR*	*FUS*	*IGK*	*LPP*	*MYD88*	*NUP98*	*PML*	*RB1*	*SSX1*	*TNFRSF13B*
ABL1	*BCL6*	*CD96*	*CSF2RA*	*EPS15*	*FUT8*	*IGL*	*LYL1*	*MYH11*	*NUTM1*	*POU2AF1*	*RBM15*	*SSX2*	*TNFRSF14*
ABL2	*BCL7A*	*CDC25A*	*CTLA4*	*ERBB2*	*GAS7*	*IKZF1*	*LYN*	*MYH9*	*NUTM2A*	*PPAT*	*REL*	*SSX4*	*TNFSF4*
ACSL6	*BCL9*	*CDK5RAP2*	*CTNNB1*	*ERG*	*GATA1*	*IL16*	*LZTS1*	*MYO18A*	*OMD*	*PPP1CB*	*RET*	*STAT3*	*TNKS2*
AFAP1L1	*BCOR*	*CDK6*	*CXCR4*	*ETS1*	*GATA2*	*IL21R*	*MAF*	*NACA*	*P2RY8*	*PRDM1*	*RFTN1*	*STAT5B*	*TOP1*
AFDN	*BCR*	*CDKN2A*	*CYB5R2*	*ETV1*	*GLI1*	*IL3*	*MAFB*	*NBEAP1*	*PABPC4*	*PRDM16*	*RHOA*	*STAT6*	*TP53*
AFF1	*BIRC3*	*CDKN2B*	*DDIT3*	*ETV3*	*GLIS2*	*IL3RA*	*MAL*	*NCOA2*	*PACSIN2*	*PRKAR2B*	*RHOH*	*STIL*	*TP63*
AFF4	*BLNK*	*CDKN2C*	*DDX10*	*ETV4*	*GMPS*	*IRAG2*	*MALT1*	*NDRG1*	*PAFAH1B2*	*PRKCA*	*RNF11*	*STRBP*	*TPM3*
AICDA	*BMF*	*CDX2*	*DDX6*	*ETV5*	*GPHN*	*IRF4*	*MAML3*	*NEK6*	*PAICS*	*PRKCB*	*RNF213*	*STRN*	*TPM4*
AKT3	*BMP7*	*CEBPD*	*DEK*	*ETV6*	*H4C13*	*IRF8*	*MDS2*	*NF1*	*PAX3*	*PRKCD*	*RNF217-AS1*	*SYK*	*TRA*
ALK	*BRAF*	*CEBPE*	*DENND3*	*EWSR1*	*HERPUD1*	*ITK*	*MECOM*	*NF2*	*PAX5*	*PRRX1*	*ROS1*	*TAF15*	*TRAF1*
ARHGAP26	*BRCA1*	*CEBPG*	*DLEU1*	*EXOC2*	*HEY1*	*ITPKB*	*MEF2B*	*NFKB1*	*PAX7*	*PSIP1*	*RPL22*	*TAL1*	*TRAF2*
ARHGEF12	*BRCA2*	*CEP43*	*DLEU2*	*EZH2*	*HIP1*	*JAK1*	*MEF2D*	*NFKB2*	*PBX1*	*PTCH1*	*RPN1*	*TAL2*	*TRB*
ARID1A	*BTG1*	*CHIC2*	*DLEU7*	*FAM216A*	*HLF*	*JAK2*	*MIGA1*	*NFKBIE*	*PCLAF*	*PTEN*	*RUNX1*	*TARBP1*	*TRD*
ARNT	*BTK*	*CHN1*	*DNMT3A*	*FBXW7*	*HMGA1*	*JAK3*	*MLF1*	*NIN*	*PCM1*	*PTK2B*	*RUNX1T1*	*TBL1XR1*	*TRG*
ASB13	*CAMTA1*	*CIC*	*DNMT3B*	*FCGR2B*	*HMGA2*	*JAZF1*	*MLLT1*	*NME1*	*PCSK7*	*PTK7*	*RUNX2*	*TCF3*	*TRIM24*
ASXL1	*CARD11*	*CIITA*	*DNTT*	*FCRL4*	*HOXA11*	*KAT6A*	*MLLT10*	*NOTCH1*	*PDCD1*	*PTPN1*	*S1PR2*	*TCL1A*	*TRIP11*
ATF1	*CARS1*	*CKS1B*	*DUSP22*	*FEV*	*HOXA13*	*KDSR*	*MLLT3*	*NOTCH2*	*PDCD1LG2*	*PTPRD*	*SART3*	*TCL1B*	*TTL*
ATG5	*CBFA2T3*	*CLIP3*	*DUX4*	*FGFR1*	*HOXA3*	*KIF5B*	*MLLT6*	*NPM1*	*PDE4DIP*	*PYCR1*	*SEC31A*	*TEC*	*TYK2*
ATIC	*CBFB*	*CLP1*	*E2F2*	*FGFR2*	*HOXA9*	*KLF2*	*MME*	*NR4A3*	*PDGFB*	*RAB29*	*SERPINA9*	*TET1*	*USP6*
ATM	*CBL*	*CLTC*	*EBF1*	*FGFR3*	*HOXC11*	*KMT2A*	*MN1*	*NRAS*	*PDGFRA*	*RABEP1*	*SET*	*TET2*	*WT1*
B2M	*CCDC50*	*CLTCL1*	*EGFR*	*FIP1L1*	*HOXC13*	*KMT2D*	*MNX1*	*NRG1*	*PDGFRB*	*RAD51B*	*SF3B1*	*TFE3*	*XPO1*
BARD1	*CCND1*	*CNTRL*	*EIF4A1*	*FLI1*	*HOXD11*	*KRAS*	*MRTFA*	*NSD1*	*PER1*	*RAF1*	*SH3BP5*	*TFG*	*YPEL5*
BATF3	*CCND2*	*COL1A1*	*EIF4A2*	*FLT3*	*HOXD13*	*LAIR1*	*MSI2*	*NSD2*	*PHF1*	*RAG1*	*SH3GL1*	*TFPT*	*ZBTB16*
BAX	*CCND3*	*CPSF6*	*ELF4*	*FNBP1*	*HSP90AA1*	*LASP1*	*MSN*	*NSD3*	*PICALM*	*RAG2*	*SLC1A2*	*TFRC*	*ZMYM2*
BCL10	*CCP110*	*CREB3L1*	*ELL*	*FOXO1*	*HSP90AB1*	*LCP1*	*MTCP1*	*NTRK1*	*PIM1*	*RALGDS*	*SNX29*	*TLX1*	*ZNF384*
BCL11A	*CD274*	*CREB3L2*	*ELN*	*FOXO3*	*ID3*	*LIMD1*	*MUC1*	*NTRK2*	*PIM2*	*RANBP1*	*SOCS1*	*TLX3*	*ZNF501*
BCL11B	*CD28*	*CREBBP*	*EML4*	*FOXO4*	*IDH1*	*LMNA*	*MYB*	*NTRK3*	*PLAG1*	*RANBP2*	*SRSF3*	*TMPRSS2*	
BCL2	*CD44*	*CRLF2*	*ENTPD1*	*FOXP1*	*IDH2*	*LMO1*	*MYBL1*	*NUMA1*	*PLCG1*	*RAP1GDS1*	*SS18*	*TNFAIP3*	
BCL2A1	*CD79A*	*CSF1*	*EP300*	*FSTL3*	*IGH*	*LMO2*	*MYC*	*NUP214*	*PLCG2*	*RARA*	*SSBP2*	*TNFRSF11A*	
